# Supplementary material for: Novel QTLs for salinity tolerance revealed by genome-wide association studies of biomass, chlorophyll and tissue ion content in 176 rice landraces from Bangladesh
Source: PLoS One. 2021 Nov 5;16(11):e0259456. doi: 10.1371/journal.pone.0259456 (PMC8570475; doi:10.1371/journal.pone.0259456)
Supplement: S1 Text — (DOCX) [file pone.0259456.s001.docx]

**Gene Expression and Functional Studies**

**Please refer to the git repository for the tables and images mentioned in the text in these notes.

**Methods:**

Gene annotations were collected from the MSU7 and RAP-DB databases. RNA-seq expression data for 12 tissues and 4 seedlings were collected from MSU7. The sequence reads had been mapped by TopHat and the RNA-seq libraries had been calculated with Cufflinks. RNA-seq data for one salt sensitive variety, Vialone Nano and one tolerant variety, Baldo were sourced from supplementary table 2 of Formentin et al. 2018 [1]. Raw reads were normalized using DESeq2 to enable between-sample comparisons. Prior to visualization, the expression table had been filtered under the criteria that every row sum value is greater than 1 and log transformed by the function $f\left( x \right)={log}_{2}(x+1)$. Genes having a p-value < 0.01 between their normalized read counts under control and stress conditions were considered significant for differential expression. Genome-wide LD decay was plotted using PopLDdecay. Heatmaps were generated using pyplot functions from the Matplotlib library in Python. For hierarchical clustering, the hclust and heatmap functions in R were used. An elaborate pipeline written in Python and R for the determination of SNP substitution effects is documented in GitHub. One-way ANOVA and t-tests were conducted using the SciPy library. The Benjamini-Hochberg FDR correction was conducted using the statsmodels library in Python.

**Results:**

**Gene expression profiling and candidate gene selection**

To select a set of informative candidate genes with authentic functional roles, we collected plant tissue-specific gene expression data from the MSU7 database and differential gene expression data under control and salt stress conditions in one sensitive variety, Vialone Nano and one tolerant variety, Baldo from a previous study [1]. The differential expression under control and stress conditions had been quantified separately in the roots and leaves. We visually profiled the expression signature of all genes located within the defined range of the 13 identified QTLs. Very sparsely expressed genes had to be removed to reduce skewness and aid visualization. A total of 278 candidate genes from within the 13 QTLs that remained after pruning of poorly expressed genes by their raw FPKM read counts can be found in supplementary table 10. Differential expression data for these genes collected from Formentin et al. 2018 [1] have been normalized and compiled in supplementary table 11.

From the MSU7 data, eight genes were found to have very high relative expression levels and have been included in table 3 as outliers in gene expression. The genes associated with stress tolerance are markedly more expressed across all tissues and have particularly higher expression in the seedlings. A cluster map for these genes has been plotted in supplementary figure 7 (a). The salinity tolerance related genes could be designated to 4 expression groups and 10 sub-clusters based on their expression levels in different tissues. To group candidate genes by their inter-tissue expression signature, we clustered genes having similar gene expression profiles into 10 groups by hierarchical clustering. Genes belonging to the same cluster will display similar relative expression patterns across all tissues. 77 genes exhibit significant differential expression across control and stress conditions in either the sensitive or the tolerant variety. A heatmap showing the differential expression of these genes in roots and leaves of the sensitive and tolerant variety has been plotted in supplementary figure 7 (b). These 77 genes have been included in table 3 as differentially expressed genes in control and stress conditions. A peroxidase precursor protein from qCDP6.1, Class III peroxidase 88 (prx88) (MSU ID: LOC_Os06g35480), an unannotated gene from qCDP12.3, LOC_Os12g41550 and a cellulose synthase like-gene from qCDP7.1, OsCSLF (MSU ID: LOC_Os07g36740) vary greatly in their expression patterns in the salt sensitive and tolerant variety (Supplementary figure 7 (b)). Six other genes, OsSGT1 (MSU ID: LOC_Os09g34250), LOC_Os06g35630, OsBGLU22 (MSU ID: LOC_Os05g30350), LOC_Os03g03630, OsIAP100 (MSU ID: LOC_Os10g35030) and LOC_Os12g06190 are expressed with moderate variation in the sensitive and tolerant variety in response to salt stress (Table 3).

**Haplotype testing and candidate gene curation**

Out of all SNP markers, we extracted SNPs including indels that occur within the 278 candidate genes. 23064 possible marker-transcript pairs within candidate gene regions were determined, and the consequence of the substitution at the polymorphic allele was elucidated. The complete table of SNP effects including the aligned transcript, site of alteration (exon/intron), occurrence of frameshift and consequence of substitution (synonymous, missense, nonsense) is provided in supplementary table 12.

For each substitution, we tested for significant difference of means between the observed genotypes using One-way ANOVA and the student’s t-test. In One-way ANOVA, three genotype groups were denoted AA, Aa and aa defined as those having two, one and no substituted alleles respectively and for the student’s t-test, only homozygotes were tested. Here, we ignore the confounding caused by population structure and carry out general hypothesis testing as these candidate genes all belong to QTLs that were originally identified using protocols that adjust for confounding with necessary corrections. Since the presence of extreme observations could bias the results for one genotype group, instead of using all the observed phenotype values, we used a filtered subset of values for each phenotype from the information previously presented in supplementary table 6. FDR correction for multiple testing was conducted by the Benjamini-Hochberg method. The substitutions showing a significant difference in the means of their observed phenotypes at an FDR adjusted p value cutoff of 0.01 along with their corresponding phenotype effects can be found in supplementary tables 13 and 14 for One-way ANOVA and the student’s t-test respectively. QQ-plots for p values from One-way ANOVA and the t-test are shown in supplementary figure 7.

Transcripts having nonsense codons in the exon region are more likely to be functionally impaired but only variants having frameshift effects that are not towards the very end of the coding sequence are certain to have lost some biological function. Under this model of thought, we sought to identify single polymorphisms having the most consequential functional effect at the protein level. Out of 23064 substitution events within 13 QTLs, we found 22 total frameshift and nonsense consequences. The summary statistics for these 22 SNPs belonging to 16 genes along with traits where significant differences were observed is presented in table 4. There are six common genes between tables 3 and 4, namely, OsPRX86 (MSU ID: LOC_Os06g35520), OsDH1 (MSU ID: LOC_Os08g43190), LOC_Os08g43370, LOC_Os09g15389, LOC_Os09g19160 and OsPIL11 (MSU ID: LOC_Os12g41650). Two of these common genes, LOC_Os09g15389 and LOC_Os09g19160 are differentially expressed under control and stress conditions. The common genes are of interest as they exhibit a significant difference in phenotype as a result of deleterious SNP substitutions in addition to functional annotations and differential expression patterns.

**Table 3: Reviewed candidate genes**

| QTL | Locus ID | Functionally annotated | Outlier in expression | Differentially expressed between control and stress | Varying expression pattern between sensitive and tolerant | MSU7 Annotation |
| --- | --- | --- | --- | --- | --- | --- |
| qCDP1.1 | LOC_Os01g65920 | Yes | No | No | N/A | F-box/LRR-repeat protein 2, putative, expressed |
| qCDP1.1 | LOC_Os01g66000 | Yes | No | Yes | No | NADH dehydrogenase I subunit N, putative, expressed |
| qCDP1.1 | LOC_Os01g66050 | Yes | No | Yes | No | wound-responsive family protein, putative, expressed |
| qCDP1.1 | LOC_Os01g66100 | Yes | No | Yes | No | gibberellin 20 oxidase 2, putative, expressed |
| qCDP1.1 | LOC_Os01g66120 | Yes | No | No | N/A | No apical meristem protein, putative, expressed |
| qCDP1.1 | LOC_Os01g66170 | No | No | Yes | No | SNARE associated Golgi protein, putative, expressed |
| qCDP1.1 | LOC_Os01g66190 | No | No | Yes | No | expressed protein |
| qCDP1.1 | LOC_Os01g66200 | No | No | Yes | No | expressed protein |
| qCDP1.1 | LOC_Os01g66300 | No | No | Yes | No | KH domain containing protein, putative, expressed |
| qCDP1.1 | LOC_Os01g66350 | Yes | No | Yes | No | DUF647 domain containing protein, putative, expressed |
| qCDP3.1 | LOC_Os03g03350 | No | No | Yes | No | polygalacturonase, putative, expressed |
| qCDP3.1 | LOC_Os03g03360 | No | Yes | No | N/A | ribosomal protein L5, putative, expressed |
| qCDP3.1 | LOC_Os03g03390 | No | No | Yes | No | G-patch domain containing protein, expressed |
| qCDP3.1 | LOC_Os03g03450 | No | No | Yes | No | anthranilate phosphoribosyltransferase, chloroplast precursor, putative, expressed |
| qCDP3.1 | LOC_Os03g03460 | Yes | No | Yes | No | erythronate-4-phosphate dehydrogenase, putative, expressed |
| qCDP3.1 | LOC_Os03g03470 | No | No | Yes | No | expressed protein |
| qCDP3.1 | LOC_Os03g03510 | Yes | No | No | N/A | CAMK_KIN1/SNF1/Nim1_like.15 - CAMK includes calcium/calmodulin depedent protein kinases, expressed |
| qCDP3.1 | LOC_Os03g03550 | No | No | Yes | No | bZIP family transcription factor, putative, expressed |
| qCDP3.1 | LOC_Os03g03590 | Yes | No | No | N/A | transporter, monovalent cation:proton antiporter-2 family, putative, expressed |
| qCDP3.1 | LOC_Os03g03600 | No | No | Yes | No | fasciclin-like arabinogalactan protein, putative, expressed |
| qCDP3.1 | LOC_Os03g03630 | No | No | Yes | Yes | expressed protein |
| qCDP3.1 | LOC_Os03g03700 | Yes | No | Yes | No | MLO domain containing protein, putative, expressed |
| qCDP3.1 | LOC_Os03g03720 | Yes | Yes | Yes | No | glyceraldehyde-3-phosphate dehydrogenase, putative, expressed |
| qCDP3.1 | LOC_Os03g03810 | Yes | Yes | Yes | No | DEF8 - Defensin and Defensin-like DEFL family, expressed |
| qCDP3.1 | LOC_Os03g04060 | Yes | No | Yes | No | CHIT16 - Chitinase family protein precursor, expressed |
| qCDP5.1 | LOC_Os05g30280 | No | No | Yes | No | Os5bglu20 - beta-glucosidase homologue, similar to G. max isohydroxyurate hydrolase, expressed |
| qCDP5.1 | LOC_Os05g30300 | No | No | Yes | No | Os5bglu21 - beta-glucosidase homologue, similar to G. max isohydroxyurate hydrolase, expressed |
| qCDP5.1 | LOC_Os05g30350 | No | No | Yes | Yes | Os5bglu22 - beta-glucosidase homologue, similar to G. max isohydroxyurate hydrolase, expressed |
| qCDP5.1 | LOC_Os05g30390 | No | No | Yes | No | Os5bglu23 - beta-glucosidase homologue, similar to G. max isohydroxyurate hydrolase, likely pseudogene in japonica, expressed |
| qCDP5.1 | LOC_Os05g30454 | No | No | Yes | No | thiamin pyrophosphokinase 1, putative, expressed |
| qCDP5.1 | LOC_Os05g30530 | Yes | No | Yes | No | 40S ribosomal protein S4, putative, expressed |
| qCDP6.1 | LOC_Os06g35410 | Yes | No | Yes | No | growth regulator related protein, putative, expressed |
| qCDP6.1 | LOC_Os06g35480 | No | No | Yes | Yes | peroxidase precursor, putative, expressed |
| qCDP6.1 | LOC_Os06g35520 | Yes | No | No | N/A | peroxidase precursor, putative, expressed |
| qCDP6.1 | LOC_Os06g35530 | Yes | No | No | N/A | CGMC_GSK.8 - CGMC includes CDA, MAPK, GSK3, and CLKC kinases, expressed |
| qCDP6.1 | LOC_Os06g35574 | Yes | No | No | N/A | mki67 protein, putative, expressed |
| qCDP6.1 | LOC_Os06g35630 | No | No | Yes | Yes | reticuline oxidase-like protein precursor, putative, expressed |
| qCDP6.1 | LOC_Os06g35650 | No | No | Yes | No | reticuline oxidase-like protein precursor, putative, expressed |
| qCDP6.1 | LOC_Os06g35670 | No | No | Yes | No | expressed protein |
| qCDP6.1 | LOC_Os06g35730 | No | No | Yes | No | ribosomal protein L5, putative, expressed |
| qCDP6.1 | LOC_Os06g36600 | Yes | No | No | N/A | retrotransposon protein, putative, unclassified |
| qCDP7.1 | LOC_Os07g36500 | No | No | Yes | No | Core histone H2A/H2B/H3/H4 domain containing protein, putative, expressed |
| qCDP7.1 | LOC_Os07g36544 | No | No | Yes | No | serine/threonine-protein kinase receptor precursor, putative, expressed |
| qCDP7.1 | LOC_Os07g36600 | Yes | No | Yes | No | universal stress protein domain containing protein, putative, expressed |
| qCDP7.1 | LOC_Os07g36740 | Yes | No | Yes | Yes | CSLF4 - cellulose synthase-like family F; beta1,3;1,4 glucan synthase, expressed |
| qCDP7.1 | LOC_Os07g37030 | Yes | Yes | No | N/A | cytochrome b6-f complex iron-sulfur subunit, chloroplast precursor, putative, expressed |
| qCDP7.1 | LOC_Os07g37210 | Yes | No | No | N/A | MYB family transcription factor, putative, expressed |
| qCDP7.1 | LOC_Os07g37240 | Yes | Yes | No | N/A | chlorophyll A-B binding protein, putative, expressed |
| qCDP7.1 | LOC_Os07g37610 | Yes | No | No | N/A | B3 DNA binding domain containing protein, putative, expressed |
| qCDP7.1 | LOC_Os07g37640 | Yes | No | No | N/A | IKI3 family protein, expressed |
| qCDP8.1 | LOC_Os08g43130 | Yes | No | No | N/A | NAP1, putative, expressed |
| qCDP8.1 | LOC_Os08g43170 | No | No | Yes | No | hydroxymethylglutaryl-CoA synthase, putative, expressed |
| qCDP8.1 | LOC_Os08g43180 | No | No | Yes | No | expressed protein |
| qCDP8.1 | LOC_Os08g43190 | No | Yes | No | N/A | dehydrogenase, putative, expressed |
| qCDP8.1 | LOC_Os08g43200 | Yes | No | No | N/A | dehydration-responsive element-binding protein, putative, expressed |
| qCDP8.1 | LOC_Os08g43230 | No | No | Yes | No | TraB family protein, putative, expressed |
| qCDP8.1 | LOC_Os08g43270 | No | No | Yes | No | BAG domain-containing protein, putative, expressed |
| qCDP8.1 | LOC_Os08g43300 | No | No | Yes | No | UBX domain-containing protein, putative, expressed |
| qCDP8.1 | LOC_Os08g43320 | No | No | Yes | No | OsRhmbd14 - Putative Rhomboid homologue, expressed |
| qCDP8.1 | LOC_Os08g43350 | No | No | Yes | No | NC domain-containing protein, putative, expressed |
| qCDP8.1 | LOC_Os08g43370 | Yes | No | No | N/A | 6-phosphogluconolactonase, putative, expressed |
| qCDP8.1 | LOC_Os08g43410 | Yes | No | No | N/A | LRP1, putative, expressed |
| qCDP9.1 | LOC_Os09g15330 | No | No | Yes | No | transporter family protein, putative, expressed |
| qCDP9.1 | LOC_Os09g15365 | Yes | No | No | N/A | hydrophobic protein, putative, expressed |
| qCDP9.1 | LOC_Os09g15370 | No | No | Yes | No | expressed protein |
| qCDP9.1 | LOC_Os09g15389 | No | No | Yes | No | expressed protein |
| qCDP9.1 | LOC_Os09g15420 | Yes | No | Yes | No | NAD dependent epimerase/dehydratase family protein, putative, expressed |
| qCDP9.1 | LOC_Os09g15520 | Yes | No | No | N/A | oleosin, putative, expressed |
| qCDP9.2 | LOC_Os09g18450 | No | No | Yes | No | flavonol synthase/flavanone 3-hydroxylase, putative, expressed |
| qCDP9.2 | LOC_Os09g19160 | No | No | Yes | No | serine/threonine-protein kinase, putative, expressed |
| qCDP9.2 | LOC_Os09g19229 | No | No | Yes | No | protein kinase domain containing protein, expressed |
| qCDP9.3 | LOC_Os09g34180 | Yes | No | Yes | No | formin, putative, expressed |
| qCDP9.3 | LOC_Os09g34250 | No | No | Yes | Yes | UDP-glucoronosyl and UDP-glucosyl transferase domain containing protein, expressed |
| qCDP9.3 | LOC_Os09g34300 | Yes | No | Yes | No | Inositol 1, 3, 4-trisphosphate 5/6-kinase, putative, expressed |
| qCDP9.3 | LOC_Os09g34310 | No | No | Yes | No | expressed protein |
| qCDP9.3 | LOC_Os09g34320 | No | No | Yes | No | expressed protein |
| qCDP9.3 | LOC_Os09g34340 | No | No | Yes | No | expressed protein |
| qCDP9.3 | LOC_Os09g34920 | No | No | Yes | No | glycosyl hydrolase family 29, putative, expressed |
| qCDP9.3 | LOC_Os09g34960 | No | No | Yes | No | hydroxymethylglutaryl-CoA synthase, putative, expressed |
| qCDP10.1 | LOC_Os10g34680 | Yes | No | No | N/A | vacuolar protein sorting-associated protein 52, putative, expressed |
| qCDP10.1 | LOC_Os10g34790 | No | No | Yes | No | terpene synthase, putative, expressed |
| qCDP10.1 | LOC_Os10g34920 | Yes | No | Yes | No | secretory protein, putative, expressed |
| qCDP10.1 | LOC_Os10g34930 | Yes | No | Yes | No | secretory protein, putative, expressed |
| qCDP10.1 | LOC_Os10g35010 | No | No | Yes | No | ATTIC110/TIC110, putative, expressed |
| qCDP10.1 | LOC_Os10g35020 | No | No | Yes | No | glycosyltransferase, putative, expressed |
| qCDP10.1 | LOC_Os10g35030 | Yes | Yes | Yes | Yes | IAP100, putative, expressed |
| qCDP10.1 | LOC_Os10g35050 | No | Yes | No | N/A | aquaporin protein, putative, expressed |
| qCDP10.1 | LOC_Os10g35070 | No | No | Yes | No | alpha-galactosidase precursor, putative, expressed |
| qCDP10.1 | LOC_Os10g35110 | Yes | No | No | N/A | alpha-galactosidase precursor, putative, expressed |
| qCDP10.1 | LOC_Os10g35180 | Yes | No | No | N/A | white-brown complex homolog protein 11, putative, expressed |
| qCDP12.1 | LOC_Os12g06020 | No | No | Yes | No | mRNA-decapping enzyme, putative, expressed |
| qCDP12.1 | LOC_Os12g06180 | No | No | Yes | No | HVA22, putative, expressed |
| qCDP12.1 | LOC_Os12g06190 | No | No | Yes | Yes | expressed protein |
| qCDP12.1 | LOC_Os12g06330 | Yes | No | No | N/A | CPuORF6 - conserved peptide uORF-containing transcript, expressed |
| qCDP12.2 | LOC_Os12g28100 | Yes | No | No | N/A | NBS-LRR disease resistance protein, putative, expressed |
| qCDP12.2 | LOC_Os12g28250 | Yes | No | No | N/A | disease resistance protein RPM1, putative, expressed |
| qCDP12.2 | LOC_Os12g28260 | Yes | No | No | N/A | cyclic nucleotide-gated ion channel, putative, expressed |
| qCDP12.3 | LOC_Os12g41380 | Yes | No | Yes | No | ulp1 protease family protein, putative, expressed |
| qCDP12.3 | LOC_Os12g41500 | Yes | No | Yes | No | rhodanese-like domain containing protein, putative, expressed |
| qCDP12.3 | LOC_Os12g41550 | No | No | Yes | Yes | expressed protein |
| qCDP12.3 | LOC_Os12g41590 | No | No | Yes | No | oxidoreductase, short chain dehydrogenase/reductase family domain containing protein, expressed |
| qCDP12.3 | LOC_Os12g41600 | Yes | No | Yes | No | OsSAUR57 - Auxin-responsive SAUR gene family member, expressed |
| qCDP12.3 | LOC_Os12g41650 | Yes | No | No | N/A | helix-loop-helix DNA-binding domain containing protein, expressed |
| qCDP12.3 | LOC_Os12g41680 | Yes | No | Yes | No | No apical meristem protein, putative, expressed |
| qCDP12.3 | LOC_Os12g41690 | No | No | Yes | No | membrane associated DUF588 domain containing protein, putative, expressed |
| qCDP12.3 | LOC_Os12g41700 | Yes | No | Yes | No | LSD1 zinc finger domain containing protein, expressed |
| qCDP12.3 | LOC_Os12g41720 | Yes | No | Yes | No | PLA IIIA/PLP7, putative, expressed |

**Table 4: Frameshift and nonsense mutants**

| QTL | MID | ID | AA, Aa, aa | ANOVA FDR adjusted p value | t test FDR adjusted p value | Marker LD (r2) | Mean LD of QTL | Significant differences observed in | Transcript | Frameshift | Effect | Expression cluster | MSU7 Annotation |
| --- | --- | --- | --- | --- | --- | --- | --- | --- | --- | --- | --- | --- | --- |
| qCDP6.1 | 10154 | 7535187 | 124,2,50 | - | 1.59E-06 | 0.258674094 | 0.12676456 | Stress shoot length, SES, shoot length, shoot weight | LOC_Os06g35520.1 | yes | nonsense | 1 | peroxidase precursor, putative, expressed |
| qCDP7.1 | 11478 | 8902226 | 89,7,80 | 0.0032397 | 0.00197096 | 0.112924056 | 0.091756821 | Root length | LOC_Os07g36570.1 | no | nonsense | 2 | KI domain interacting kinase 1, putative, expressed |
| qCDP8.1 | 13576 | 10399469 | 125,4,47 | 0.01677336 | 0.006898917 | 0.048195612 | 0.124458416 | Stress shoot length | LOC_Os08g43140.1 | no | nonsense | 9 | expressed protein |
| qCDP8.1 | 14044 | 10400946 | 171,1,4 | - | 0.000400579 | 0.077104648 | 0.124482397 | Shoot length | LOC_Os08g43190.1 | no | nonsense | 5 | dehydrogenase, putative, expressed |
| qCDP8.1 | 14235 | 10401783 | 132,2,42 | - | 0.002544629 | 0.061615259 | 0.124469548 | Shoot length, stress shoot length | LOC_Os08g43240.1 | yes | nonsense | 8 | LTPL97 - Protease inhibitor/seed storage/LTP family protein precursor, expressed |
| qCDP8.1 | 15501 | 10404138 | 154,0,22 | - | 0.00408966 | 0.048884575 | 0.124458987 | Root length | LOC_Os08g43370.1 | yes | nonsense | 9 | 6-phosphogluconolactonase, putative, expressed |
| qCDP8.1 | 15505 | 10404142 | 149,1,26 | - | 0.007674301 | 0.040686999 | 0.124452187 | Stress shoot weight | LOC_Os08g43370.1 | yes | nonsense | 9 | 6-phosphogluconolactonase, putative, expressed |
| qCDP9.1 | 16280 | 10899825 | 175,0,1 | - | 0.006134065 | 0.346305387 | 0.275364277 | Shoot weight, SES, shoot length | LOC_Os09g15389.2 | yes | nonsense | 1 | expressed protein |
| qCDP9.1 | 16759 | 10904247 | 137,3,36 | - | 0.001980025 | 0.472905577 | 0.27557995 | Shoot weight, shoot length | LOC_Os09g15530.1 | yes | missense | 5 | expressed protein |
| qCDP9.1 | 16787 | 10904278 | 143,1,32 | - | 0.002128613 | 0.383739751 | 0.275428049 | Shoot weight, shoot length | LOC_Os09g15530.1 | yes | nonsense | 5 | expressed protein |
| qCDP9.1 | 16795 | 10904286 | 146,1,29 | - | 0.003161695 | 0.344007186 | 0.275360362 | SES, shoot length, shoot weight, stress shoot length | LOC_Os09g15530.1 | yes | nonsense | 5 | expressed protein |
| qCDP9.1 | 16879 | 10905149 | 136,2,38 | - | 0.000788829 | 0.483012624 | 0.275597168 | Shoot length, shoot weight, shoot thickness | LOC_Os09g15570.1 | no | nonsense | 2 | OsFBX315 - F-box domain containing protein, expressed |
| qCDP9.2 | 17727 | 11026378 | 110,9,57 | 0.00523674 | 0.002837199 | 0.157495672 | 0.081696535 | Lost root length | LOC_Os09g19160.1 | yes | missense | 2 | serine/threonine-protein kinase, putative, expressed |
| qCDP9.2 | 17504 | 11025985 | 107,10,59 | 0.00648358 | 0.002863092 | 0.166716977 | 0.081717831 | Lost root length | LOC_Os09g19160.1 | no | nonsense | 2 | serine/threonine-protein kinase, putative, expressed |
| qCDP10.1 | 20747 | 12341326 | 145,3,28 | - | 0.000400579 | 0.036083425 | 0.048629414 | Shoot length | LOC_Os10g34820.1 | no | nonsense | 1 | CDT1B - Putative DNA replication initiation protein, expressed |
| qCDP10.1 | 21242 | 12344280 | 92,4,80 | 0.00938081 | 0.003717159 | 0.103744084 | 0.048691346 | Root length | LOC_Os10g34960.1 | no | nonsense | 5 | ubiquitin family protein, putative, expressed |
| qCDP10.1 | 21304 | 12344780 | 168,1,7 | - | 0.008595073 | 0.052325971 | 0.048644281 | Stress shoot sodium content | LOC_Os10g35000.1 | no | nonsense | 1 | G-patch domain containing protein, expressed |
| qCDP12.2 | 24817 | 14757369 | 171,0,5 | - | 0.009590229 | 0.021513239 | 0.163924811 | Stress root length | LOC_Os12g27994.2 | no | nonsense | 1 | expressed protein |
| qCDP12.2 | 24872 | 14761966 | 147,2,27 | - | 0.000813891 | 0.142509545 | 0.164209508 | Lost root length, stress shoot length | LOC_Os12g28065.1 | no | nonsense | 1 | expressed protein |
| qCDP12.3 | 26887 | 15177683 | 172,0,4 | - | 0.008366562 | 0.25745731 | 0.208912404 | Stress chlorophyll B content | LOC_Os12g41650.3 | no | nonsense | 1 | helix-loop-helix DNA-binding domain containing protein, expressed |
| qCDP12.3 | 26723 | 15177683 | 111,2,63 | - | 0.008366562 | 0.25745731 | 0.208912404 | Stress chlorophyll B content | LOC_Os12g41650.4 | no | nonsense | 1 | helix-loop-helix DNA-binding domain containing protein, expressed |

References:

1. Formentin, E., et al., *Transcriptome and Cell Physiological Analyses in Different Rice Cultivars Provide New Insights Into Adaptive and Salinity Stress Responses.* Frontiers in Plant Science, 2018. **9**(204).
