# Supplementary figures and images for: Novel QTLs for salinity tolerance revealed by genome-wide association studies of biomass, chlorophyll and tissue ion content in 176 rice landraces from Bangladesh
Source: PLoS One. 2021 Nov 5;16(11):e0259456. doi: 10.1371/journal.pone.0259456 (PMC8570475; doi:10.1371/journal.pone.0259456)

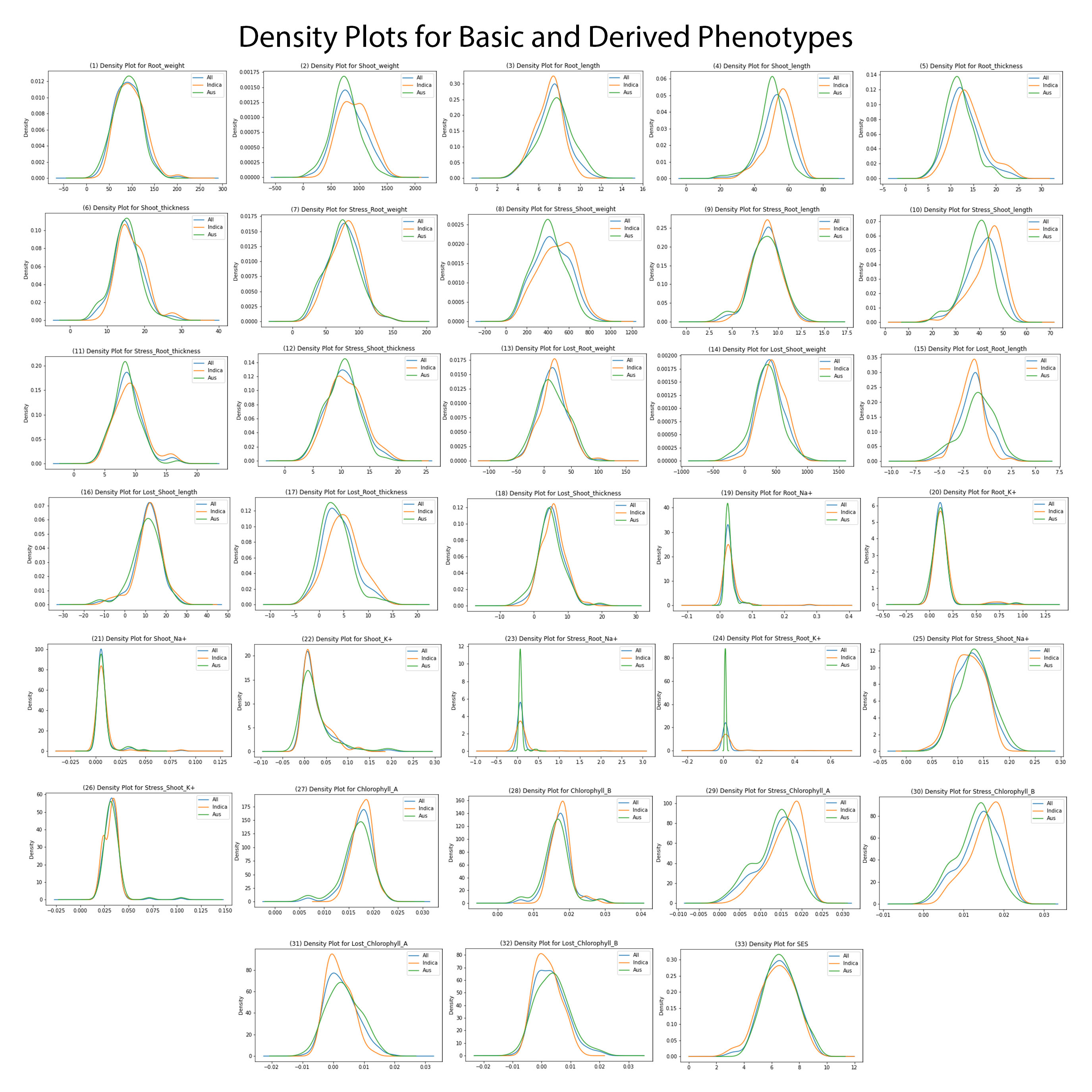

Supplement: S2 Fig — (JPG) [file pone.0259456.s003.jpg]

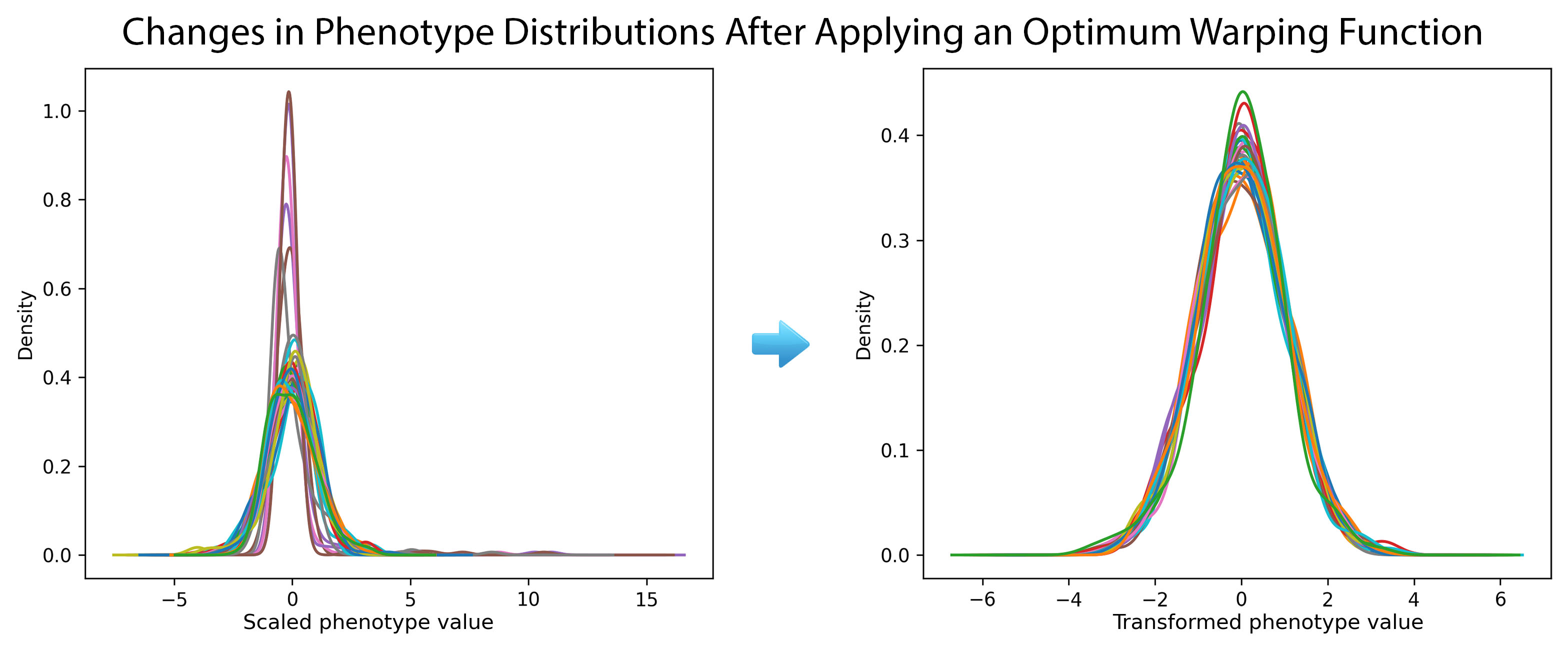

Supplement: S3 Fig — (JPG) [file pone.0259456.s004.jpg]

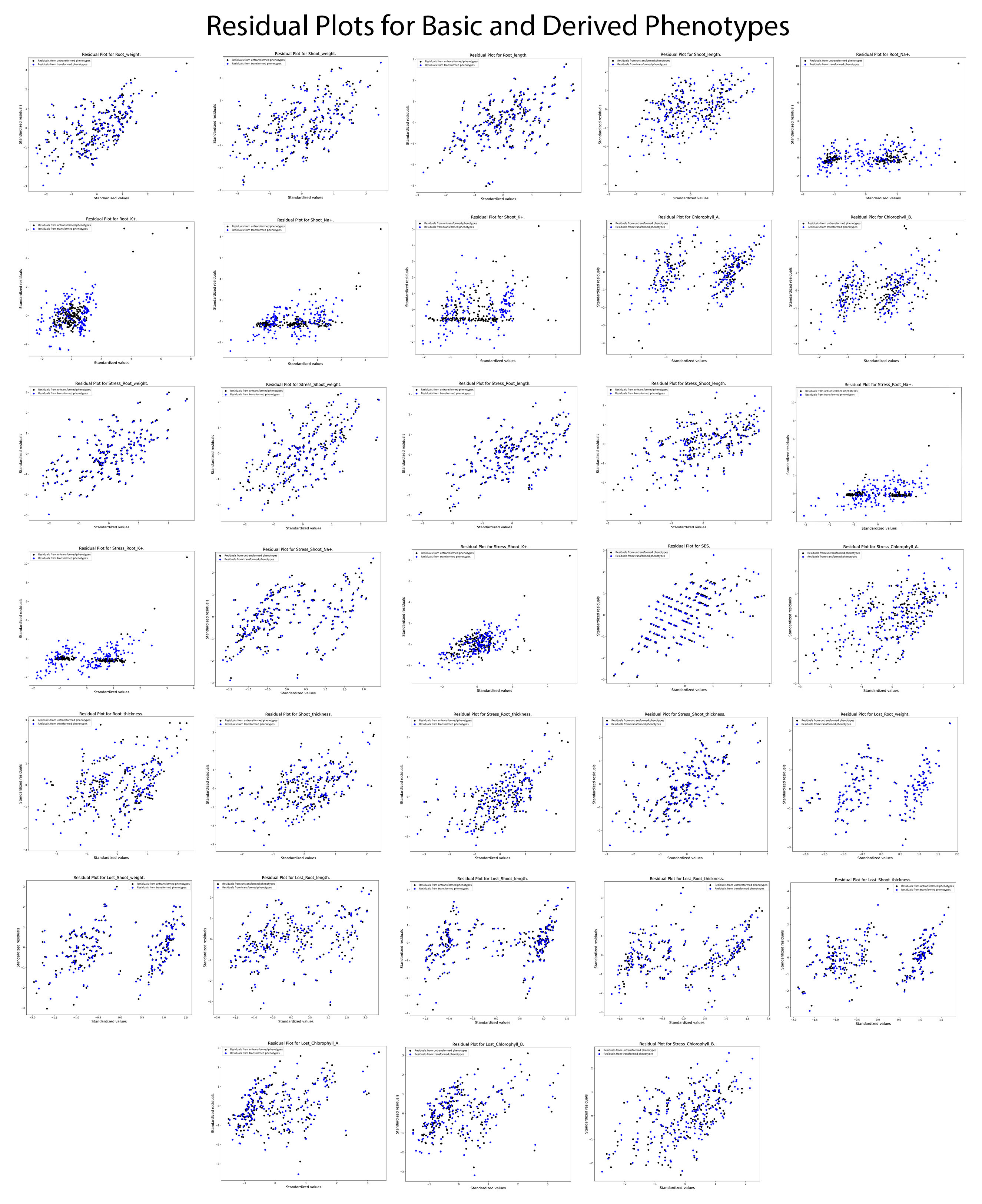

Supplement: S4 Fig — (JPG) [file pone.0259456.s005.jpg]

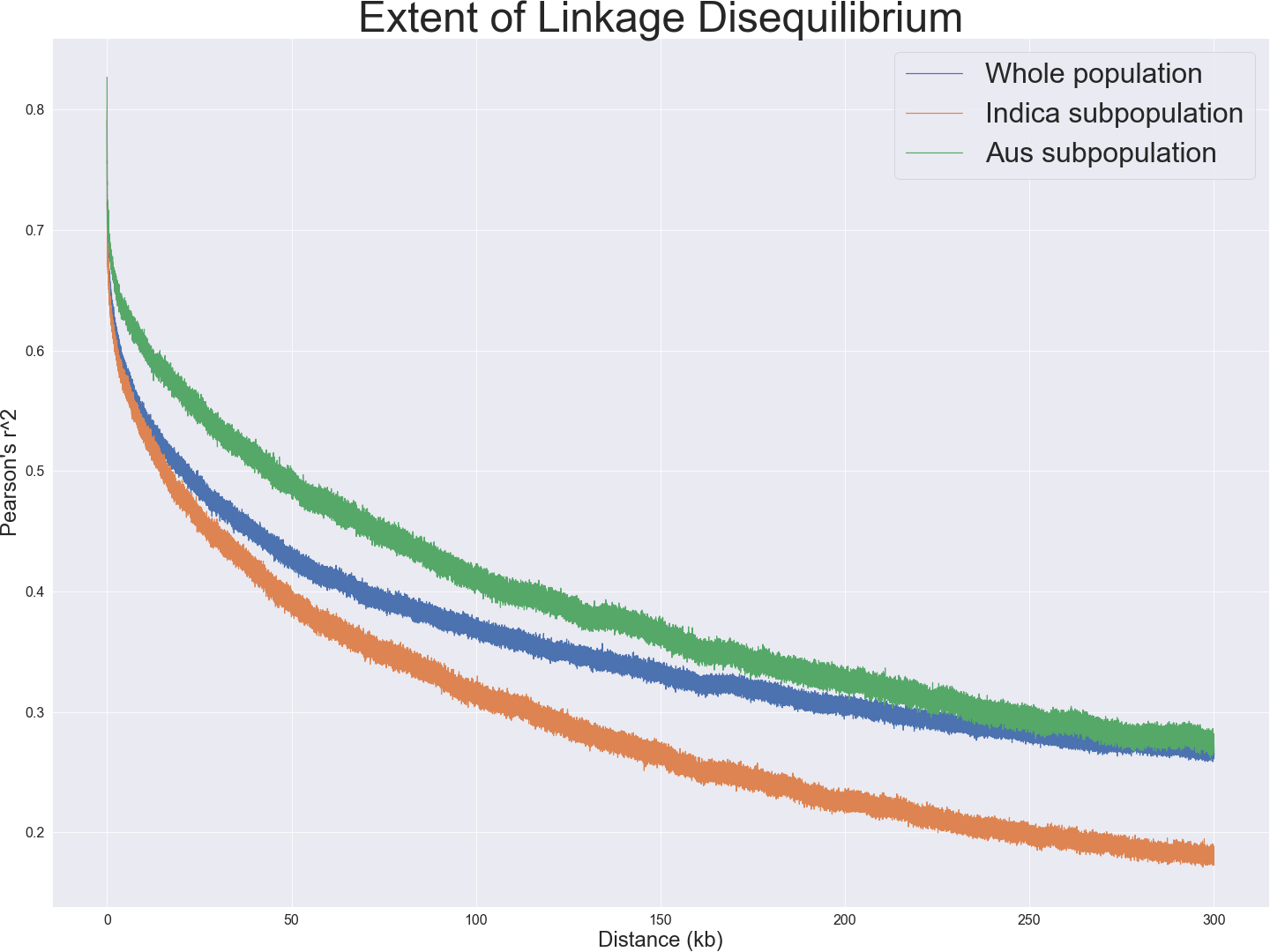

Supplement: S6 Fig — (JPG) [file pone.0259456.s007.jpg]
