# Supplementary material for: Novel QTLs for salinity tolerance revealed by genome-wide association studies of biomass, chlorophyll and tissue ion content in 176 rice landraces from Bangladesh
Source: PLoS One. 2021 Nov 5;16(11):e0259456. doi: 10.1371/journal.pone.0259456 (PMC8570475; doi:10.1371/journal.pone.0259456)
Supplement: Attachment — Submitted filename: Response to reviewers 2.docx [file pone.0259456.s024.docx]

Reviewer #1:

Comment: The manuscript by Alam et al. now titled “Novel QTLs for salinity tolerance revealed genome-wide association studies of biomass, chlorophyll and tissue ion content in 176 rice landraces from Bangladesh” is a revised version of a previously submitted paper. I describes a GWAS mapping analysis of 11 phenotypes yielding 13 QTL and list of associated candidate genes.

The manuscript has been substantially edited and most of my concerns were addressed. However, there are still issues that need to be addressed to improve the quality of this manuscript.

Line 28: take out “sophisticated”. This is a qualified personal opinion and not a quantified fact. There are still a number of such embellishing qualifiers that need to be removed (see below).

Response: Removed in line 28.

Comment: Line 63: take out “elaborately”. This is an opinion.

Response: Removed in line 62.

Comment: Line 68: edit to “…publicly available gene expression profiles….”, because you did not do this for the current paper.

Response: Added in line 67.

Comment: Line 211: take out “elaborate” (see comments above).

Response: Section has been removed.

Comment: Lines 235-238: use past tense, not present tense, to describe your results.

Response: New lines 233-234 have been changed to past tense.

Comment: Lines 252-253: change sentence to “We observed better trait correlations under salt stress conditions than control conditions”. The “fortification” term is too confusing.

Response: Changed in line 250.

Comment: Lines 264-28): I still think this paragraph is too long and has unnecessary information. Simply state that non-Gaussian distributions were normalized before GWAS analyses.

Response: We have kept the information about data distributions, the test for normalcy and rationale for transformation. We have removed everything else. The new section is in lines 260-273.

Comment: Line 281: remove “extensive”. Again, this is an unnecessary qualification/personal opinion.

Response: Removed in line 292.

Comment: Line 292: spell out CDP (Comprehensive Data for Plants). Actually, my recommendation is to change the naming to something reflecting salts stress since CDP is a very idiosyncratic.

Response: The original dataset generated in our laboratory, from which data was used to prepare the first version of this manuscript, has been titled as such. The authors plan to carry out more GWAS studies with their data using the computation pipeline that has been established in this manuscript. The authors wish to maintain this naming convention for the sake of consistency and convenience.

Comment: Lines 295-296: remove the sentence starting with “The suggestive association…”. There is no follow-up to the statement.

Response: Sentence was removed in line 307.

Comment: Line 304: change “connotations” to “evaluations”.

Response: The authors feel that the word ‘connotations’ is more appropriate here.

Comment: Lines 330-331: change “imparts sufficient prospect in the role…” to “suggest a role..”.

Response: Changed in line 344.

Comment: Line 241: change “To select a set of informative candidate genes with authentic functional roles..” to simply “To select a set of candidate genes…”. The rest are unnecessary qualifications.

Response: Section has been moved to supplementary notes. So, left unchanged.

Comment: Line 425: change “traditionalist” to “traditional”.

Response: Changed in line 377.

Comment: Line 428: remove “deliberately”.

Response: Removed in line 380.

Comment: Lines 431-433: modify the sentence starting with “The lack of…” to “The lack of this correlation in shoots under stress conditions suggests that sodium exclusion from rice leaves might be critical for survival in salt stress”. At the point, a correlation is not an “elegant demonstration” without further analyses to “demonstrate” causation.

Response: The authors concede. The change has been made in lines 384-285.

Comment: Line 437: remove “highly”.

Response: Changed in line 389.

Comment: Line 457: change “causing a nonsense consequence” to “generating a stop codon”.

Response: Section has been moved to supplementary.

Comment: Line 477: remove the first part of the sentence (“With rigorous….significant thresholds”) and start sentence simply with “We mapped…”.

Response: Changed in line 426.

Comment: Line 494: remove “greatly”.

Response: Changed in line 443.

Reviewer #3:

Comment: The paper has merit and acceptable for publication. However, it needs major revision and should be shortened to focus on QTL detection for salinity tolerance by GWAS. The candidate gene extraction by outsourced RNASeq data is highly speculative in relation to QTLs detected in this study.

Response: We appreciate the new perspectives and valuable insights from the reviewer. In this revision, we omit outsourced expression studies from the main manuscript and add more detail about inter-variety variations in Bangladeshi landraces in response to salt stress.

Comment: RNAseq Data were outsourced from MSU, while Vialone Nano and Baldo RNAseq data were from Formentin 2018. Gene expression and downstream analysis is not appropriate to be included in this paper because RNA gene expression is 1.) genotype-specific, 2.) tissue-specific, 3.) developmental stage-specific, and most importantly, 4.) treatment-specific. While you may have the same ID or variety, the available RNA expression data is only specific to the materials they tested during that time. Moreover, RNA expression studies needs further validation because RNAs are unstable and transient naturally. For this study, to outsource RNASeq data is not acceptable because even MSU RNA Seq data were not collected during salinity stress of the test samples, but mostly during developmental stage of rice and tissue-specific.

Response: The reviewer raises some valid concerns. We agree that the impact of the manuscript up to QTL mapping is far greater than its remainder which yields speculative conclusions at best. We have streamlined the manuscript further to accommodate the views of the reviewer. Instead of completely deleting the expression analysis and functional consequence studies, we have moved the complete section to supplementary notes should any keen reader be interested in going over them.

Comment: Scientific papers are inherently difficult to read and authors should strive to make it more comprehensible by using more simple words to encourage readers. For example line 253-261 states “Rectangle C1 and S1 compare correlations between biomass traits in control and stress conditions, C2 and S2 compare correlations between chlorophyll, SES and biomass in control and stress conditions. C3 and S3 compare correlations between chlorophyll and SES in control and stress. The improved linear correlations between biomass, chlorophyll and SES traits imply that the encumbrance of salt stress on a plant prevents the disproportionate gain of biomass. Since plant biomass and chlorophyll content is markedly reduced under salt stress (supplementary table 3), we can conclude that salt stress does not affect all phenotypes for all genotypes at the same rate and the genetic advantages that any genotype has in terms of tissue growth (root/shoot) or chlorophyll accumulation will be compromised by the effect of abiotic stress at a greater magnitude than traits which have near-baseline values.”

The correlations of traits do not mean causation or effects, it is simply telling a trend between the two traits. Why is such a long confusing conclusion here? What exactly the authors would like to convey based on correlations? It’s a fact that traits like biomass and chlorophyll content are affected by salt stress! The paper would be more meaningful if they measured the reduction of traits in control and stressed genotypes. It would be interesting to find out which varieties had been least affected by the stress and by how much, compare to other genotypes, or an analyses like some sort of index of growth reduction comparison among genotypes with regards to stress and control treatments.

Response: The reviewer is correct that correlations of traits do not explicitly mean causation or effects but we disagree with their opinion that correlation is ‘simply a trend’. The word ‘trend’ in statistics is more appropriate for fields like economics where only directional patterns are observed.

We want to take this opportunity to break down the section quoted here by the reviewer. To make things simple, let us look at the correlation between Shoot weight and Chlorophyll A in control and stress conditions. Please download the notebook at: [https://github.com/DeadlineWasYesterday/Cat-Does-Plant/blob/master/Figures%20and%20tables/Revision%202%20argument.pdf](about:blank) to follow this demonstration. There should be a download button on the top right of the preview in this webpage.

Please look at figure 1 in this notebook. It shows that shoot weight is reduced under salt stress. Figure 2 shows a less dramatic, but identifiable reduction in chlorophyll A content. From this, we can conclude that shoot weight and chlorophyll A content is reduced under salt stress. Figures 3 and 4 linearly model the nature of this reduction. We can observe a clear linear relationship under stress conditions which was less prominent in the absence of salt. This is what the color depth of the rectangles in figure 3 of our manuscript signifies.

In figure 5 we have plotted the distribution of the ratio of shoot weight and chlorophyll A content from supplementary table 3. This shows that both the magnitude and the spread of the shoot weight to chlorophyll A ratio is reduced in stress conditions. The bidirectional bar chart found in the last figure (out[10]), shows a dramatic difference between the dispersal of shoot weight to chlorophyll A ratios in our experimental varieties. From this bar chart, we can see that plants that had a high ratio in control conditions, exhibit smaller ratios in stress conditions.

This is the exact conclusion we have drawn in the section quoted from the manuscript by the reviewer. The correlation heatmap on figure 3, the p values in supplementary table 5 and the density plots in supplementary figure 2 directly demonstrate this phenomenon. To paraphrase: *Salt does not affect all genotypes equally. In salt stress, phenotypes correlate better linearly and the ratios become more proportional, which is to say that any advantage that a genotype has in terms of disproportionate tissue growth or chlorophyll accumulation will be compromised first under salt stress in general.*

We beg the reviewer to not undermine our analyses by calling them ‘merely a trend’. A sufficient amount of thought and attention was put into this. The authors feel that there is ample data in the manuscript to support their claim.

We have added lines 263-275 and a new figure 4 to bring forward the effects of salt stress on phenotypes of different varieties.

Comment: The QTL naming is not reflecting of the association of a trait to a loci. The CDP is too broad and not meaningful to associate to salinity tolerance or any specific trait.

Response: The rationale behind the original naming convention was that this was a diverse set of phenotype data for GWAS analysis. We had filtered out quite a few before writing the original manuscript and as per the recommendations of the reviewers, traits other than those concerned with salinity tolerance were left out from the first revision. The naming is consistent throughout the manuscript, images and supplementary files and we hope to keep them consistent in our following work using data that have been excluded from this manuscript. Additionally, the QTL names with qCDP are hardcoded in all the sourcecode, making our experiments easily reproducible using data that was made available online.

Comment: In Discussions, “Biomass traits and chlorophyll content could be valuable indices for the screening of salinity tolerance”—this is already known fact.

Response: There are a number of recent studies on salinity tolerance where the experimental design exclusively focuses on ion content and ion ratios and/or overlooks simpler observations such as biomass and chlorophyll content [1-3]. The authors agree that the effects of salt stress on biomass and chlorophyll is quite obvious. In spite of this, our study as a whole brings forward the numerical relationships between these phenotypes in response to salinity tolerance. The quoted section in particular reflects the basic impression of our journey throughout the study. A lot of it builds on the referenced manuscript by Pires et al. 2015 [4]. The authors wish to keep the section as is.

Comment: Please state the criticism/problem to SES that needs to be addressed. Line 407-408 is intriguing or dramatic and maybe unnecessary to be written.

Response: SES is a composite score which is prone to human error. The principal component we derived from stress biomass and chlorophyll content in this revision would be more numerically robust and a better index for salt injury than an evaluation made by the naked eye. Of course, SES scoring is less laborious and still applicable in many scenarios. The authors feel that a comprehensive study of salt tolerance will still be incomplete without recording biomass and chlorophyll content. In light of this, we have appended the following section at the end of our conclusions in line 435: “Although it is well established that biomass and chlorophyll related traits alongside tissue ion content aids the assessment of salinity tolerance, our studies show that the numerical values of plant biomass and chlorophyll content could manifest themselves into a more objective index of salt injury unabated by human error.”

Comment: In discussion, again, high and low correlations are not causation or effects but simply statistical trends. It should also be noted if the correlations are negative or positive. Authors should refrain from drawing too much generalization or speculations unless supported with hard proof validation especially that this study is dealing with diverse germplasms.

Response: The blue color in figure 3 and supplementary figure 1 indicates a positive correlation and the red color indicates negative. Out of the significant correlations shown in figure 3, only SES shares negative correlation with the remaining phenotypes. The authors have addressed part of this comment in detail in the response to the second comment.

Comment: Discussion 4.1 is too general and mostly known fact by previous studies. The paper would be more meaningful if authors discussed the traits of varieties used in response to salt stress. This study is nothing new except for the varieties used, so it would be nice to have those information available to the readers in comparison to other known salinity tolerance study.

Response: The authors feel differently about section 4.1. We feel that the contents of the section have more depth than the reviewer has surmised here. The distributions of our phenotypes have been observed. We have discussed why we did not derive phenotypes from sodium and potassium ratios, which are very commonly used in the screening of salinity tolerance. We feel that our study puts forward ample evidence to allow us to emphasize the value of biomass and chlorophyll data in the study of salinity tolerance.

Comment: The paper is claiming novel QTLs, therefore, the focus of discussion should be those novel QTLs, their significance, effects- positive or negative effect, enhancing tolerance or sensitivity, variance explained by the QTLs, occurrence, and usefulness in future salt tolerance introgression. Authors may further discuss the similarity and differences of detected QTLs in relation to previous other QTLs.

Response: This is primarily a QTL detection and mapping study. Molecular dissection of these QTLs requires thorough and extensive laboratory work, which the authors have been planning for the near future. The topics brought up by the reviewer are very relevant and will be the subject of our subsequent studies.

Comment: In Discussion 4.2, Lines 452-455, candidate genes discussed in the paper were selected on the basis of (a) functionally impairment of gene in the population and (b) individuals carrying the genotype of the functional allele are quantifiably different in terms of phenotype. The study would be meaningful and therefore has merit to discuss candidate genes in details if candidate genes were re-sequenced to confirm the functional SNPs, and validated by gene expression like qRT-PCR. At this point, candidate genes as underlying genes controlling salinity tolerance is premature assumptions despite employing extensive statistical analysis. Gene expression is study-specific and should be tested in contrasting genotypes.

Response: These sections have been removed in accordance with the comments from the reviewer.

Comment: The paper is too lengthy for the methods, and discussion was too short to emphasize the actual findings of the study. While extensive data mining was conducted, the claims regarding candidate genes are not fully supported by current findings. Further validation is needed. However, if the paper is shorted into QTL detection alone, the study is meritorious of publication. I suggest to remove the candidate gene mining by RNASEq, unless fully supported by re-sequencing, functional SNP study and gene expression analysis. We uphold to scientific and rigorous standard regardless of negative or positive results. It is no excuse to say that this study started out as a student project and that it is time constraints and with limited budget. However, once published, this paper would stand as scholastic achievement and pride of the student and all authors included.

Response: We have revised the manuscript as per the suggestions of the reviewer.

References:

1.	Warraich, A.S., et al., *Rice GWAS reveals key genomic regions essential for salinity tolerance at reproductive stage.* Acta Physiologiae Plantarum, 2020. **42**(8): p. 134.

2.	Kumar, V., et al., *Genome-wide association mapping of salinity tolerance in rice (Oryza sativa).* DNA Research, 2015. **22**(2): p. 133-145.

3.	Batayeva, D., et al., *Genome-wide association study of seedling stage salinity tolerance in temperate japonica rice germplasm.* BMC Genetics, 2018. **19**(1): p. 2.

4.	Pires, I.S., et al., *Comprehensive phenotypic analysis of rice (Oryza sativa) response to salinity stress.* Physiologia Plantarum, 2015. **155**(1): p. 43-54.
