# Supplementary material for: Novel QTLs for salinity tolerance revealed by genome-wide association studies of biomass, chlorophyll and tissue ion content in 176 rice landraces from Bangladesh
Source: PLoS One. 2021 Nov 5;16(11):e0259456. doi: 10.1371/journal.pone.0259456 (PMC8570475; doi:10.1371/journal.pone.0259456)
Supplement: Attachment — Submitted filename: Response to editor.docx [file pone.0259456.s025.docx]

Comment from the editor:

1. Since there are some associations between genes and QTLs mentioned in section 3.4 and conclusion section include a sentence 'Supplementary analysis of gene expression and functional annotations assumes potential roles for a number of genes within the identified QTLs', authors are advised to include few lines in the discussion section regarding this.

2. It seems there are some supplementary tables and figures not cited in the text. Please ensure that all supplementary information is cited.

Response:

1. We have added lines 424 to 452 regarding the expression and functional studies.

2. We have cited all the supplementary information in the text:
Supplementary table 1 cited in line 81.
Supplementary table 2 cited in line 236.
Supplementary table 3 cited in line 237.
Supplementary table 4 cited in line 246.
Supplementary table 5 cited in line 249.
Supplementary table 6 cited in line 283.
Supplementary table 7 cited in line 318.
Supplementary table 8 cited in line 327.
Supplementary table 9 cited in line 329.
Supplementary table 10 cited in line 427.
Supplementary table 11 cited in line 430.
Supplementary table 12 cited in line 432.
Supplementary table 13 cited in line 433.
Supplementary table 14 cited in line 434.
Supplementary figure 1 cited in line 248.
Supplementary figure 2 cited in line 277.
Supplementary figure 3 cited in line 287.
Supplementary figure 4 cited in line 289.
Supplementary figure 5 cited in line 307.
Supplementary figure 6 cited in line 324.
Supplementary figure 7 cited in line 430.
Supplementary text 1 cited in line 426.
